# Resurgence of Type III IGR Between I73R and I329L in Wild Boars With African Swine Fever in South Korea in 2023

**DOI:** 10.1155/tbed/2524367

**Published:** 2025-01-03

**Authors:** Garam Kim, So-Jeong Kim, Jung-Hyeuk Kim, Yong-Kwan Kim, Weon-Hwa Jheong

**Affiliations:** Wildlife Disease Response Team, National Institute of Wildlife Disease Control and Prevention (NIWDC), Gwangsan-gu, 1 Songam-gil, Gwangju, Republic of Korea

## Abstract

The African swine fever virus (ASFV) causes African swine fever (ASF), a highly lethal disease affecting domestic pigs and wild boars. Since its initial outbreak in October 2019 in Yeoncheon, Gyeonggi Province, ASF has continued to spread in South Korea. This study aimed to differentiate closely related ASFV strains through the analysis of the intergenic region (IGR) between *I73R* and *I329L* genes. In 2019, genetic analysis confirmed one IGR I type case and two IGR III type cases in Paju, followed by two more IGR III type cases in 2020. After a period of detecting only the IGR II type, IGR III type cases re-emerged in Pohang and Cheong-song in November and December 2023. Genetic analysis using *B646L*, *CP204*, *B602L*, and *EP402R* genes confirmed that the IGR III strains belong to genotype II and serogroup 8, similar to the Georgia/2007/1 strain but differing in IGR type. Since the first occurrence of ASF in wild boars in South Korea, we have continuously monitored the introduction and variation of ASFV. As a result, we reconfirmed the presence of the IGR III type ASFV in 2023, 3 years and 8 months later, in a different area from where it was last detected. This finding would not have been possible without the continuous monitoring of ASFV introduction and genetic variation. We emphasize the critical role of regular monitoring based on molecular markers and comprehensive genomic analysis in enhancing the effectiveness of ASFV control and prevention.

## 1. Introduction

African swine fever virus (ASFV) is a highly contagious, enveloped, double-stranded DNA virus, belonging to the Asfarviridae family, that causes African swine fever (ASF), a severe hemorrhagic disease, with up to 100% mortality rates in both domestic pigs and wild boars [1, 2]. The ASFV genome is composed of a linear, double-stranded DNA of 170–190 kbp, characterized by terminal inverted repetitions and hairpin loops [3, 4]. Genomic sequence analysis has demonstrated considerable conservation in the central region of the genome. However, the genomic ends display significant variability, particularly within the left 40 kbp and right 15 kbp regions, providing important molecular insights into strain differentiation [5–8].

Genetic characterization of ASFV has been a focus of recent studies, particularly through the partial sequencing of the gene encoding the major capsid protein p72 (*B646L*). To date, 24 distinct genotypes of ASFV have been identified through the sequencing of p72, making it a key marker for molecular polymorphisms [9]. Further improvement in intragenotypic resolution has been achieved through the sequencing of the *E183L* gene, which encodes p54, allowing for a more refined understanding of ASFV phylogeny [10]. Phylogenetic analyses based on p54 and p72 sequences reveal fundamental similarities; however, additional subdivision within the primary branch of p72 genotype I has been observed, leading to the identification of four subclusters, designated as Ia, Ib, Ic, and Id [8, 11]. In addition to *B646L* and *E183L*, the *CP204L* gene, which encodes the structural protein p30, has been utilized to further explore molecular differences among ASFV strains, particularly to assess evolutionary trends within geographically distinct isolates [12–14]. Previous studies have emphasized the value of using a combination of the *B646L*, *E183L*, and *CP204L* genes for strain characterization, highlighting its superiority over the need for multiple additional markers [6].

ASFV, like its counterparts of large, nucleocytoplasmic DNA viruses, houses a central variable region (CVR) spanning ~400 bp, nestled within the consistently stable, evolutionarily conserved core of 125 kbp [15]. The *B602L* gene within the CVR region acts as an important variable genetic indicator, demonstrating its ability to differentiate viruses sharing identical p72 and p54 genotypes [16]. Sequencing of this gene has identified 31 viral subgroups with varying tetrameric amino acid repeats, making it a particularly discriminative genetic marker [17]. Moreover, the EP402R gene, which has been used to identify eight ASFV serotypes, adds further layers to ASFV characterization [18, 19]. Further genetic differentiation of closely related ASFV isolates has been achieved through the analysis of specific intergenic regions (IGRs), such as *I73R*/*I329L* and *I78R*/*I215L*, which serve as markers for genetic variation [8, 17]. In the *I73R*/*I329L* region, specific genetic patterns, such as “GGAATATATA,” have been used to classify ASFV into four distinct types [20]. Examination of these IGRs has been instrumental in differentiating closely related ASFV strains during outbreaks [21]. Comparative analysis of the genetic characteristics of specific regions of ASFV has proven to be useful in elucidating the origin and transmission pathways of ASFV during ASF outbreaks [22]. According to reports, genetic analyses based on sequences of *B646L*, *B602L*, *EP402R*, and IGR were conducted during ASFV outbreaks in China, Russia, Estonia, and Uganda to analyze the epidemiological situation [23–26].

This study focuses on the genetic analysis of ASFV strains found in wild boars in South Korea between 2019 and 2023, particularly the IGR between *I73R* and *I329L*. In 2023, the re-emergence of IGR III type in two regions marked its first detection since 2020. The study highlights the importance of tracking ASFV evolution, especially new IGR types, which could indicate viral adaptations or cross-border transmission. Continuous monitoring and genetic characterization of ASFV in South Korea are essential for controlling outbreaks, and understanding its genetic diversity contributes to future molecular epidemiological studies.

## 2. Materials and Methods

### 2.1. Analysis of the Distribution of IGR Type

From 2019 to 2023, we examined the occurrence of ASF and utilized open-source Geographic Information System software version 3.24 (www.qgis.org, accessed on July 25, 2023) to explore the spatial distribution of these outbreaks. Subsequently, a cartographic analysis was performed using geographical coordinates associated with each ASF outbreak.

### 2.2. Detection of ASFV in Wild Boar Samples

DNA was extracted from wild boar blood using the Maxwell RSC Viral Total Nucleic Purification Kit (Promega, Madison, WI, USA) according to the manufacturer's instructions. The presence of ASFV DNA was detected using polymerase chain reaction (PCR) with the following ASF diagnostic primers: PPA1 (5′-AGTTATGGGAAACCCGACCC-3′), PPA2 (5′-CCCTGAATCGGAGCATCCT-3′) [27], P72D (5′-GTACTGTAACGCAGCACAG-3′), and P72U (5′-GGCACAAGTTCGGACATGT-3′) [11]. The VetMAX ASFV Detection Kit, commercially available from Thermo Fisher Scientific in the United States, was used according to the manufacturer's guidelines, utilizing 5 µL of DNA. ASFV was detected in the FAM channel, whereas the internal positive control was detected in the VIC channel of the instrument [28]. These primers partially amplified the *B646L* (p72) region.

### 2.3. PCR Amplification and Sequencing of Targeted Genes

PCR amplification of ASFV was performed using the primers listed in Table 1. The target genes were amplified via PCR using a BioFACT 2X Multi-Star PCR series DNA polymerase kit (BioFACT, Daejeon, South Korea). All *B646L*, *EP402*, *B602L*, *CP204L*, and *I73R/I329L* amplicons were sequenced using the corresponding PCR primers in separate reactions at annealing temperatures of 60, 52, 58, 53, and 56°C, respectively. All PCR products were run on a 1.5% agarose gel and sized against a 1 kb DNA plus ladder (BioFACT, Daejeon, South Korea). We commissioned BioFACT (Daejeon, South Korea) to analyze the purification and Sanger sequencing of the amplified products.

### 2.4. Characterization of ASFV and Phylogenetic Analysis

Sequences were aligned using multiple alignments within a fast Fourier transform algorithm in Geneious Prime version 2023.0.4 (accessed on July 25, 2023) to determine the phylogenetic relationship of ASFV. Subsequently, a maximum likelihood (ML) phylogenetic tree was constructed for genetic regions between *I73R/I329L*, *B646L*, *CP204L*, and *EP402R* using the ClustalW in Molecular Evolutionary Genetics Analysis (MEGA) X to evaluate the support for the phylogenetic tree [31]. A total of 1000 replicates of ML bootstrapping were performed to assess the robustness of tree topologies.

## 3. Results

### 3.1. Distribution of IGR by Type From 2019 to 2023 in South Korea

**Figure 1:**
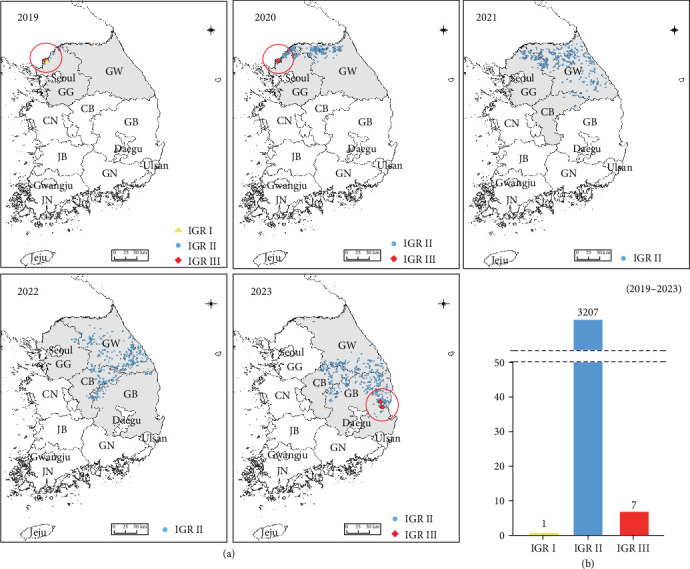
Analysis of the IGR types occurring in Korea from 2019 to 2023. (a) IGR results on a map based on the year. (b) The number of IGR types identified from 2019 to 2023. IGR I is represented in yellow, IGR II in blue, and IGR III in red. The red circle indicates the location of a newly-discovered IGR type. CB, Chungcheongbuk-do; CN, Chungcheongnam-do; GB, Gyeongsangbuk-do; GG, Gyeonggi-do; GN, Gyeongsangnam-do; GW, Gangwon-do; IGR, intergenic region; JB, Jeollabuk-do; JN, Jeollanam-do.

ASF outbreaks in wild boars in South Korea were initially detected in October 2019, with 3495 positive cases confirmed by 2023. The diagnosis was conducted using the VetMAX ASFV Detection Kit and ASFV-specific primer targeting the *B646L* and *PPA*. In particular, the *B646L* and PPA are well-known for reflecting the genetic characteristics of ASFV and are conserved regions that allow for differentiation from other viruses, making them suitable targets for diagnosis [11, 27]. Additionally, IGR analysis between *I73R* and *I329L* was performed to distinguish genotypes. In 2019, in Paju, one case of IGR I type and one case of IGR III type were confirmed for the first time. In March 2020, two more cases of the IGR III-type were identified in Paju. Subsequently, from 2021 to 2022, only IGR II type was detected. However, in November 2023, the IGR III type was confirmed again, with two cases each in Cheongsong and two cases in Pohang (Figure 1a). From 2019 to 2023, the confirmed cases of IGR types in wild boars were one case of IGR I type, seven cases of IGR III type, and 3207 cases of IGR II type (Figure 1b).

### 3.2. IGR Analysis Between *I73R* and *I329L*

**Figure 2:**
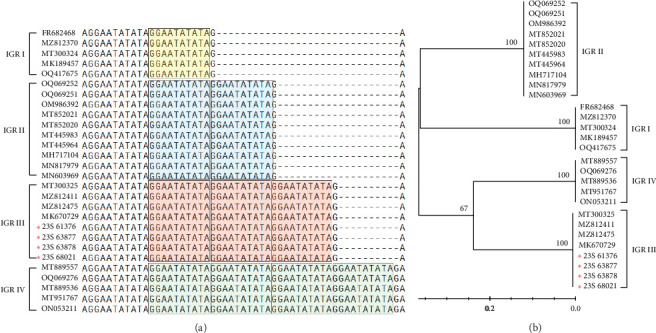
Analysis of IGR types based on gene sequence alignment and phylogenetic relationship. Nucleotide alignment of Korean ASFV strains and previously published strains with IGR sequences. (a) Dashes indicate the absence of nucleotide sequences. Strains belonging to IGR I, II, III, and IV are shown in yellow, blue, orange, and green, respectively. (b) This study focused on analyzing the gene sequences of *I73R* and *I329L* in ASFVs. By incorporating data from this study, marked by red asterisks, evolutionary relationships were elucidated using the Kimura 2-parameter model. Phylogenetic reconstruction was executed using 1000 bootstrap replications to gauge node support, with support values presented as percentage bootstrap support. Red asterisks represent Korean strains of the IGR III type confirmed in the 2023 study. ASFV, African swine fever virus; IGR, intergenic region.

The IGR between *I73R* and *I329L* is classified based on the number of repetitions of the 10-nucleotide sequence “GGAATATATA.” IGR I (two copies), IGR II (three copies), IGR III (four copies), and IGR IV (five copies) [20]. In this study, we confirmed that in the 23S-61376, 23S-63877, 23S-63878, and 23S-68021 strains, an additional insertion of the “GGAATATATA” sequence, compared to that in the IGR II type, was identified (Figure 2a). We analyzed the phylogenetic relationships of samples, including the IGR III type, by constructing a phylogenetic tree. This analysis confirmed that the IGR III strain identified in a previous study is identical to the ASFV strain detected in 2019 (GenBank accession no. MT300325). Additionally, it was confirmed that these strains belong to the IGR III type, similar to strains from Vietnam and China (GenBank accession nos. MZ812411, MZ812475, and MK670729) (Figure 2b). Several nodes with bootstrap values of 100 were observed, indicating highly reliable relationships between the groups at these nodes. Specifically, the node within the IGR III cluster with a bootstrap value of 100 strongly suggests that this group exhibits a clear phylogenetic separation (Figure 2b). This indicates that the IGR III type possesses distinct phylogenetic characteristics, differentiating it from other types, and its divergence is statistically significant.

### 3.3. Phylogenetic Analysis Based on *B646L* (p72) and *CP204* (P30)

**Figure 3:**
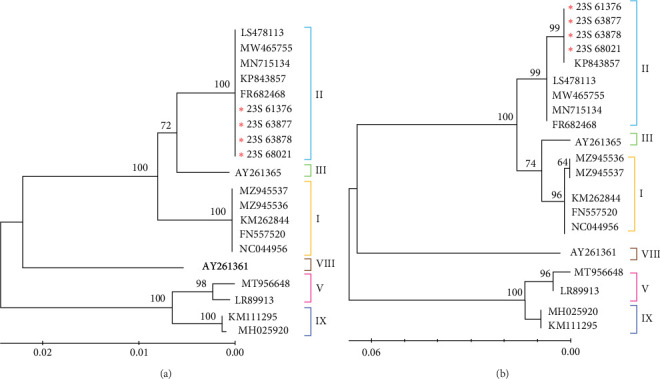
Phylogenetic trees of ASFV were constructed using nucleotide sequences from genes *B646L* (P72) (a) and *CP204L* (P30) (b). These sequences include data from the present study (represented by red asterisks). The sequences for both *B646L* (p72) and *CP204L* (P30) genes commenced with GenBank accession numbers. The Kimura 2-parameter model was used to ascertain the evolutionary history. Reconstruction of the phylogeny encompassed 1000 bootstrap replications for node support evaluation, with values depicted as percentage bootstrap support. ASFV, African swine fever virus.

The phylogenetic tree of the IGR III type ASFV, constructed based on partial sequences of the *B646L* (p72) gene of ASFVs registered in GenBank in this study, indicated that it belongs to genotype II (Figure 3a). Moreover, to assess the evolutionary differences between the ASFV variants, we conducted a genetic analysis based *on CP204L* (p30). Our results are consistent with the genotype classification based on *B646L* (p72) (Figure 3b). The results of the bootstrap analysis strongly suggest that the Korean IGR III type ASFV has a highly stable phylogenetic relationship with genotype II viruses and is closely related to this group. Furthermore, the IGR III strains identified in this study were analyzed based on *B646L* and *CP204* of the ASFV initially identified in wild boars in Korea. These results confirmed that these viruses were closely related in terms of genetics and genotype (data not shown).

### 3.4. Sequence Analysis of the CVR Within the *B602L* Gene

**Figure 4:**
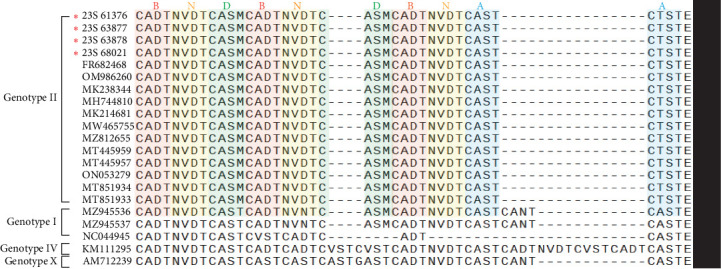
Amino acid (aa) alignment analysis based on B602L (CVR). The ASFV strains included in this study are representative, as all field strains exhibited identical nucleotide sequences. Upon analysis of the CVR amino acid sequences, tetrameric repeats were identified, and single-letter codes representing each type were assigned. The tetrameric amino acid repeats were cataloged using the following codes: A = CAST, CVST, CTST, CASI; B = CADT, CADI, CTDT, CAGT, CVDT; C = GAST, GANT; D = CASM; F = CANT, CAAT; and N = NVDT, NVGT, NVDI. ASFV, African swine fever virus; CVR, central variable region.

Specific features based on tetrameric amino acid repeats within the CVR of ASFV have been reported [17]. Analysis of the CVR of the *B602L* gene revealed that when compared with the sequences of strains of the same genotype, the IGR III strains had the CVR sequence repeated 10 times with the BNDBNDBNAA profile (Figure 4). These strains, belonging to genotype II, were grouped within the same CVR subgroup as those from Georgia, China, and Vietnam (Figure 4 and Table 2).

### 3.5. Determination of the Serogroups Based on the *EP402R* (CD2v) Gene

**Figure 5:**
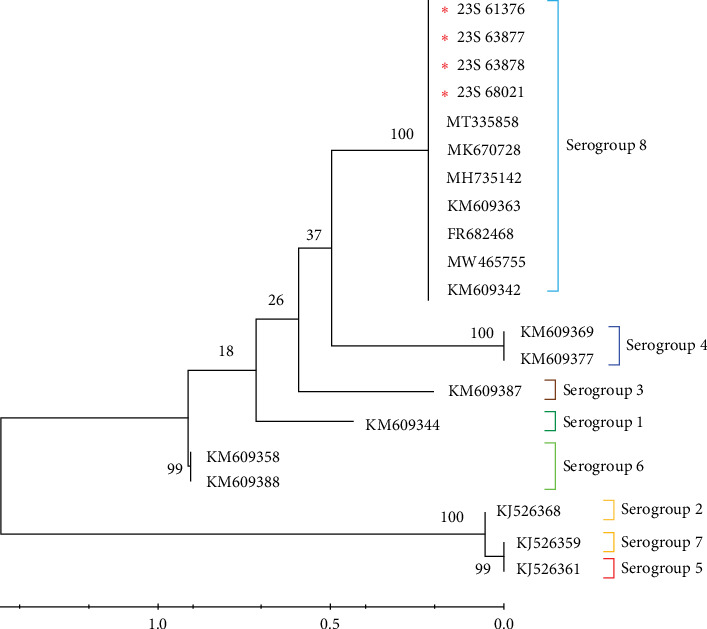
Analysis of the ASFV EP402R (CD2v) gene-based serogroups. Phylogenetic trees were constructed for ASFV using nucleotide sequences from *EP402R* (CD2V), incorporating data from this study marked by red asterisks. The evolutionary history was determined using the Kimura 2-parameter model, and phylogenetic reconstruction involving 1000 bootstrap replications was used to assess node support, expressed as percentage bootstrap support. ASFV, African swine fever virus.

ASFV strains can be classified into different serogroups based on their hemadsorption inhibition (HAI) characteristics, utilizing CD2v (*EP402R*) and/or C-type lectin (*EP153R*) [32]. To classify the strains into one of the eight serogroups in this study, representative isolates for each serogroup were compared by analyzing the sequences of *EP402R*, which encodes the CD2v major ASFV antigen protein, retrieved from GenBank. The serotype results indicated that among the compared sequences, all viruses identified in this study belonged to serotype 8 (Figure 5). The serogroup 8 containing 23S-61376, 23S-63877, 23S-63878, and 23S-68021 showed a bootstrap value of 1000, confirming that the samples within this group are closely related and form a highly reliable, independent cluster phylogenetically. This high bootstrap value suggests that these viruses share a common origin and form a genetically stable group.

## 4. Discussion

Since its first occurrence in 2019, up until 2023, 3495 cases of ASF have been reported in Korean wild boars. The whole genome of the ASFV, first detected in wild boars in 2019, was analyzed, and the introduction of the virus was investigated [33]. From 2019 to the present, annual genetic diversity analyses of the virus have been conducted by examining its genomes and genetic regions. Recently, a genetic epidemiological analysis was carried out, focusing on the regional distribution of variants identified in wild boars, including MGF 360-1L and 4L [34].

The ASFV genome is composed of linear, double-stranded DNA with sizes ranging from 170 to 190 kb, depending on mutations caused by deletions or insertions in the DNA sequence [35]. The ASFV genome consists of a central constant region of ~125 kb and two highly variable regions at each end, with the left variable region spanning 38–47 kb from the left terminus and the right variable region extending 13–16 kb from the right terminus [5, 36]. The conventional genetic subtyping strategy for ASFV analyzes the CVR of the gene, including p72 (*B646L*), p54 (*E183L*), *B602L*, and tandem repeat sequences within the IGR between *I73R* and *I329L* [11, 12]. Additionally, sequences within *p30* and CD2v (*EP402R*) have demonstrated discriminatory utility in genetic and serotype classifications [9]. The IGR distinguishes types based on the number of repeated base sequences between *I73R* and *I329L* [21, 37]. The IGR region is being used epidemiologically as a molecular marker for virus introduction and transmission associated with identified types [20, 38].

Different IGR types were observed in Paju: IGR I in December 2019 and IGR III in March 2020 (Figure 1). Subsequently, we monitored the spread of these IGR types. Following their initial detection in Paju in 2019 and 2020, the IGR types did not spread to other regions. To prevent their dissemination, fences were erected around the outbreak sites, and containment measures were intensified by capturing wild boars outside the fence to curb the spread to adjacent areas. These measures are speculated to have effectively prevented the spread of the virus to other regions. However, ~3 years and 8 months later, in 2023, the IGR III type resurfaced in Cheongsong and Pohang (Figure 1). To characterize the features of the confirmed IGR III type strains, we conducted a genetic analysis based on *B646L*, *CP204L*, *B602*, and *EP402R*. Phylogenetic analysis using sequences from *B646L*, *CP204L*, and *B602* revealed that the strain belonged to genotype II and serogroups 8, similar to the ASFV detected in Korean wild boars in 2019 and the G-2007/1 strain (Figures 3–5). However, we found that they differed only in the IGR type. Further analysis and investigation into variations in other regions will necessitate whole-genome sequencing of these viruses.

Since 2019, ASF has been persistently circulating in wild boars in South Korea. Variants of the virus circulating in specific regions have been reported. When comparing the G-2007/1 and G-2008/2 strains from Georgia, a new variation was identified in the C315R/C147L region of the G-2008/2 strain, representing the first observed repeat variation among isolates derived from the epidemic lineage [39]. In 2020, confirmation of single nucleotide polymorphism diversity in MGF 360-1La and MGF 360-4L in Korea facilitated the analysis of their geographical distribution [34]. In China, reports indicate a decrease in the virulence of the ASFV attributed to natural variations. This virus manifests mutations, insertions, and deletions, leading to amino acid alterations in 23 open reading frames [40]. According to this report, mutations in circulating viruses were unrelated to the IGR region, and as the IGR has been reported as an indicator for classifying different viruses, the reoccurrence of the IGR III is likely not the result of the variations in circulating viruses.

In this study, we confirmed the re-emergence of the IGR III type in South Korea after 3 years and 8 months since its last occurrence in 2019. According to recent reports, the IGR III type of ASFV has been detected in China and Vietnam between 2019 and 2022 [41–43]. The widespread distribution of the IGR III type suggests that it likely originated in China and subsequently spread to several other Asian countries [44, 45]. IGR serves as a useful marker for tracking genetic diversity and transmission routes, and it is used to distinguish viruses by region [45, 46]. We will continue utilizing IGR analysis to thoroughly investigate the spread patterns of ASFV, providing valuable insights into their transmission routes. Additionally, it is challenging to estimate the introduction route of the IGR III type ASFV detected in South Korea based solely on IGR analysis. Therefore, further genome analysis will be conducted to determine its potential relationship with ASFV from China and Vietnam.

## 5. Conclusions

We reconfirmed the occurrence of the IGR III type ASFV in Korean wild boars in 2023. This finding highlights the importance of continuous monitoring of ASFV introduction and genetic variation. ASF continues to spread globally, including in South Korea, with increasing reports of viral diversity. Therefore, surveillance using molecular markers and comprehensive genomic analysis is critical for effective ASFV control, prevention, and epidemiological tracing. These approaches will play a crucial role in tracking virus introductions and managing its spread in the future.

## Figures and Tables

**Table 1:** Primers used for the evaluation and amplification of ASFV.

Target		Primer sequence (5′–3′)	Tm (°C)	Reference
B646L	P72-U	GGCACAAGTTCGGACATGT	60	[11]
	P72-D	GTACTGTAACGCAGCACAG	—	—

PPA	PPA1	AGTTATGGGAAACCCGACCC	60	[27]
	PPA2	CCCTGAATCGGAGCATCCT	—	—

I73R/I329L	IGR-F	CCATTTATCCCCCGCTTTGG	56	[29]
	IGR-R	TCGTCATCCTGAGACAGCAG	—	—

B602L	CVR-F	TCGGCCTGAAGCTCATTAG	58	[30]
	CVR-R	CAGGAAACTAATGATGTTCC	—	—

CP204L	P30-F	CTGAATCTAATGAAGAAGA	53	This study
	P30-R	AAGTCTTTGTAGGTTTTTCGTTCA	—	—

EP402R	EP402R-gaF1	TACACTAGCTACATGTGGAAAAG	52	This study
	EP402R-gaR1	CTGATAACGACTGTAAGGCTTAG	—	—

Abbreviations: ASFV, African swine fever virus; CVR, central variable region; IGR, intergenic region.

**Table 2:** CVR of *B602L* within ASFV strains.

Strain	Accession no.	Country	Genotype	CVR profile	Quantity
23S-61376	This study	Korea	II	BNDBNDBNAA	10
23S-63877	This study	Korea	II	BNDBNDBNAA	10
23S-63878	This study	Korea	II	BNDBNDBNAA	10
23S-68021	This study	Korea	II	BNDBNDBNAA	10
Georgia 2007/1	FR682468	Georgia	II	BNDBNDBNAA	10
China/GX/202013	OM986260	China	II	BNDBNDBNAA	10
ASF-wbBS01	MK238344	China	II	BNDBNDBNAA	10
ASFV-SY18	MH744810	China	II	BNDBNDBNAA	10
China/Jilin/2018/boar	MK214681	China	II	BNDBNDBNAA	10
HaNam/VN2020	MW465755	Vietnam	II	BNDBNDBNAA	10
VNUA BG-ASF3	MZ812655	Vietnam	II	BNDBNDBNAA	10
VNUA TH-ASF2	MT445959	Vietnam	II	BNDBNDBNAA	10
VNUA HY-ASF3	MT445957	Vietnam	II	BNDBNDBNAA	10
VNUA HB-ASF3	ON053279	Vietnam	II	BNDBNDBNAA	10
MNG/5/BU/2019	MT851934	Mongolia	II	BNDBNDBNAA	10
MNG/3/BU/2019	MT851933	Mongolia	II	BNDBNDBNAA	10
Pig/HeN/ZZ-P1/2021	MZ945536	China	I	BNDBNDBNAA	10
Pig/SD/DY-1/2021	MZ945537	China	I	AABNAABBA	9
Ken05/Tk1	NC044945	Kenya	X	AABNAABBA	9
Ken06Bus	KM111295	Kenya	IX	ABNAAAACBNAAAAACBNAAAAACBNAAAACBNAFA	36
Benin97/1	AM712239	Benin	I	AAABNABBNABBAABNABNABA	22

Abbreviations: ASF, African swine fever; ASFV, African swine fever virus; CVR, central variable region.

## Data Availability

The data that support the findings of this study are available from the corresponding author upon reasonable request.
